# Type 2 Diabetes Mellitus Facilitates Shift of Adipose-Derived Stem Cells Ex Vivo Differentiation toward Osteogenesis among Patients with Obesity

**DOI:** 10.3390/life12050688

**Published:** 2022-05-06

**Authors:** Margarita Agareva, Iurii Stafeev, Svetlana Michurina, Igor Sklyanik, Ekaterina Shestakova, Elizaveta Ratner, Xiang Hu, Mikhail Menshikov, Marina Shestakova, Yelena Parfyonova

**Affiliations:** 1 Institute of Fine Chemical Technologies Named after M.V. Lomonosov, 119571 Moscow, Russia; amarrgo1999@gmail.com; 2 Department of Angiogenesis, National Medical Research Centre of Cardiology Named after Academician E.I. Chazov, 121552 Moscow, Russia; michurinas192@gmail.com (S.M.); eiratner@gmail.com (E.R.); myumensh@mail.ru (M.M.); yeparfyon@mail.ru (Y.P.); 3 Department of Biochemistry, Faculty of Biology, Lomonosov Moscow State University, 119991 Moscow, Russia; 4 Institute of Diabetes, Endocrinology Research Centre, 117292 Moscow, Russia; sklyanik.igor@gmail.com (I.S.); katiashestakova@mail.ru (E.S.); shestakova.mv@gmail.com (M.S.); 5 Department of Endocrinology, Union Hospital, Tongji Medical College, Huazhong University of Science and Technology, Wuhan 430074, China; huxiang628@126.com; 6 Department of Biochemistry and Molecular Medicine, Faculty of Basic Medicine, Lomonosov Moscow State University, 119991 Moscow, Russia

**Keywords:** osteogenesis, adipose-derived stem cells, obesity, insulin resistance, type 2 diabetes, cell senescence

## Abstract

Objective: Sedentary behavior with overnutrition provokes the development of obesity, insulin resistance, and type 2 diabetes mellitus (T2DM). The main progenitor cells of adipose tissue are adipose-derived stem cells (ADSCs) which can change differentiation, metabolic, and secretory phenotypes under obesity conditions. The purpose of this study was to evaluate ADSC osteogenesis activity among patients with obesity in normal glucose tolerance (NGT) and T2DM conditions. Methods: In the study, ADSCs from donors with obesity were used. After clinical characterization, all patients underwent bariatric surgery and ADSCs were isolated from subcutaneous fat biopsies. ADSCs were subjected to osteogenic differentiation, stained with Alizarin Red S, and harvested for real-time PCR and Western blotting. Cell senescence was evaluated with a β-galactosidase-activity-based assay. Results: Our results demonstrated the significantly increased calcification of ADSC on day 28 of osteogenesis in the T2DM group. These data were confirmed by the statistically significant enhancement of RUNX2 gene expression, which is a master regulator of osteogenesis. Protein expression analysis showed the increased expression of syndecan 1 and collagen I before and during osteogenesis, respectively. Moreover, T2DM ADSCs demonstrated an increased level of cellular senescence. Conclusion: We suggest that T2DM-associated cellular senescence can cause ADSC differentiation to shift toward osteogenesis, the impaired formation of new fat depots in adipose tissue, and the development of insulin resistance. The balance between ADSC adipo- and osteogenesis commitment is crucial for the determination of the metabolic fate of patients and their adipose tissue.

## 1. Introduction

Modern lifestyle and dietary habits dictate the new challenges for endocrinology and diabetology. Sedentary behavior with overnutrition provokes the development of obesity and consequent metabolic complications such as insulin resistance, dyslipidemia, cardiovascular diseases, and type 2 diabetes mellitus (T2DM) [1,2]. However, some patients with obesity do not have any metabolic abnormalities. The question about possible markers for patient predisposition to the development of metabolic complications remains open and debatable [3,4].

Adipose tissue is one of the crucial players in whole body metabolism regulation because the secretory function of adipose tissue is necessary for homeostasis [5,6]. Adipose-derived stem cells (ADSCs) have unique immune-privileged and immuno-modulating properties [7,8]. Moreover, ADSCs contain a preadipocyte fraction that controls the renewal of adipose tissue and supports normal adipose tissue physiology.

However, during pathological conditions, such as obesity and insulin resistance, ADSCs can change differentiation, metabolic, and secretory phenotypes. Some data suggest the suppression of differentiation potential and the upregulation of apoptosis and inflammation in ADSCs from patients with T2DM [9]. ADSCs from obesogenic and diabetic conditions have demonstrated the abrogation of glycolysis, OXPHOS metabolism, and the accumulation of mutations in mitochondrial DNA [10]. Moreover, ADSCs from T2DM patients have been found to exhibit lower proliferative activity and altered secretory profiles manifested as decreased VEGF, decreased adiponectin, and increased leptin secretion [11,12]. All present changes lead to metabolic-associated ADSC dysfunction and disturb the regulatory and differentiation functions of ADSCs.

According to the International Society for Cellular Therapy statement, ADSCs can be differentiated in chondrogenic, osteogenic, and adipogenic directions [13]. Nevertheless, insulin resistance and whole body energy homeostasis influence the differentiation potential of ADSC populations. High glucose concentration was found to impair differentiation and promote the stemness and expression of SOX2, OCT4, and NANOG in ADSCs [14]. Moreover, diabetic conditions are characterized by the decreased angiogenesis and differentiation of ADSCs in both human and animal models [15,16]. T2DM, especially, significantly suppresses white and beige adipogenesis [11,17,18,19]. Based on the fact that ADSC osteogenesis and adipogenesis are in a reciprocal relationship due to the mutual repression of the RUNX2 (master regulator of osteogenesis) and PPARγ (master regulator of adipogenesis) transcription factors [20,21,22], we hypothesize that the suppression of adipogenesis can be the result of activated ADSC osteogenesis in T2DM. Previous studies have confirmed increased osteogenesis [23,24], but some researchers have reported lower osteogenesis in diabetic conditions [25,26]. The purpose of present study was to evaluate ADSC osteogenesis among patients with obesity and to understand the relationships between ADSC osteo- and adipogenesis in obesogenic conditions. 

## 2. Methods and Materials

### 2.1. ADSC Isolation and Cell Culture

The study protocol was approved by the ethics committee of the Endocrinology Research Centre (Moscow, Russia) (protocol #9 from 10 May 2017). Written informed consent was obtained from each of the volunteers. Adipose tissue samples were obtained during bariatric surgery from donors with obesity and normal glucose tolerance (NGT, N = 3) or T2DM (N = 3). Anthropometric and metabolic parameters were analyzed among all donors, and the respective results are presented in Supplementary Table S1. ADSCs were isolated from subcutaneous white adipose tissue biopsies, which were minced and digested at 37 °C for 1 h with collagenase I (200 U/mL, Sigma-Aldrich, St. Louis, MO, USA) and filtered through a 100 µm cell strainer. The cells were seeded and cultured overnight in complete DMEM supplemented by 10% FBS (HyClone, Logan, UT, USA) and PenStrep followed by the exchange of the medium to remove non-adherent cells. ADSCs were incubated with 5% CO_2_ at 37 °C in the DMEM/F12 (1:1) culture medium supplemented by 10% FBS and 1% PenStrep. The media were changed every three days.

### 2.2. Osteogenic Differentiation

Osteogenic differentiation was performed according to the method of Wang et al. [27]. Briefly, ADSCs were grown up to 60% confluency, after which the culture medium was replaced by an induction medium (DMEM/F12 supplemented by 10% FBS, 1% PenStrep, 100 nM dexamethasone, 10 mM sodium-β-glycerophosphate, and 50 μM sodium ascorbate) or a control medium (DMEM/F12, 10% FBS, and 1% PenStrep). Cells were cultured with 5% CO_2_ at 37 °C, and medium replacement was performed every three days. At 14, 21, and 28 days post-induction, cells were harvested for staining, real-time PCR analysis, and Western blotting.

### 2.3. Alizarin Red S Staining

Calcium accumulation was assessed with Alizarin Red S staining according to the work of Gregory and coauthors [28]. Briefly, cells were washed three times with PBS, followed by fixation in a 4% formalin solution for 15 min at room temperature. After washing with distilled water, Alizarin Red S solution was added and incubated at room temperature for 30 min in the dark. Cells were washed five times with distilled water and visualized using phase-contrast microscopy on a Zeiss Axio Observer A1 (Zeiss, Oberkochen, Germany). The analysis of images was performed with the ZEN software (Zeiss, Germany).

For the quantification of Alizarin Red S staining, we used a spectrophotometric approach. A 10% acetic acid solution was used for the re-solubilization of dye, and samples were heated at 85 °C for 10 min with mineral oil to the top of the slurry to avoid evaporation. The slurry was centrifuged at 20,000× *g* for 15 min, and 10% ammonium hydroxide was added for acid neutralization. The absorbances of samples and Alizarin Red S standard serial dilutions were measured at 405 nm using a VICTOR X Multilabel Plate Reader (PerkinElmer, Waltham, MA, USA). The concentration of Alizarin Red S per well was calculated using the standard curve method.

### 2.4. Real-Time PCR

The mRNA levels of the crucial osteogenic markers RUNX2, SMAD4, and SOX2 were measured with real-time PCR. Total RNA was isolated from cells using the CleanRNA Standard reagent Kit (Eurogene, Moscow, Russia), according to the manufacturer’s instruction. The concentration of isolated RNA and its purity was determined with a NanoDrop2000 microvolume spectrophotometer (Thermo Scientific, Waltham, MA, USA). cDNA synthesis was performed using the RevertAid H Minus First Strand cDNA Synthesis Kit (Thermo Scientific, USA). Real-time PCR was performed using the SYBRGreen reagent kit (Synthol, Russia) on a StepOnePlus PCR system (Applied Biosystems, Waltham, MA, USA). Primers were synthesized in Eurogene, Russia; sequences are presented in Table 1.

All samples were analyzed in triplicate, and values were compared against the housekeeping gene β-actin. The mRNA level was quantified with the 2^(deltaCt) method [29]. 

### 2.5. Western Blotting

Cell lysates were separated by SDS PAGE according to the work of Laemmli [30]. After that, proteins were transferred to a PVDF membrane with consequent blocking in a 5% fat-free milk solution on TBST. Then, membranes were washed and incubated with HRP-conjugated secondary antibodies according to manufacturer instructions. The primary antibodies were: collagen I (dilution 1:1000; ab34710, Abcam, Cambridge, UK) and syndecan 1 (dilution 1:1000; ab181789, Abcam, Waltham, MA, USA); the secondary antibodies were: HRP-conjugated secondary antibody against mouse IgG (dilution 1:10,000; PA1-28748, Thermo Scientific, USA) and HRP-conjugated secondary antibody against rabbit (dilution 1:10,000; ab67216 Abcam, Waltham, MA, USA). Visualization was performed using the Clarity ECL kit (Bio-Rad, Hercules, CA, USA) and the Fusion-FX gel documentation system (Vilber Lourmat, Paris, France). Protein band analysis was performed with the GelAnalyzer2010 software. The whole blot can be found at supplementary materials (Figure S1).

### 2.6. Senescence-Associated β-Galactosidase (SA-β-Gal) Assay

Cell senescence assays were performed as previously described [31]. The medium was removed and wells were washed twice with PBS. Cells were fixed in 2% formalin and 0.2% glutaraldehyde for 5 min at room temperature. After washing, cells were stained with 1 mg/mL of X-gal in 40 mM citric acid/sodium phosphate buffer (pH 6), 5 mM potassium hexacyanoferrate ΙΙ, 5 mM potassium hexacyanoferrate ΙΙΙ, 150 mM sodium chloride, and 2 mM magnesium chloride for 16 h at 37 °C. The solution was removed and wells were washed twice with PBS, washed once with methanol, and completely dried in air. Cells were visualized using phase-contrast microscopy with a Zeiss Axio Observer A1 (Zeiss, Germany). The analysis of images was performed with the ZEN software (Zeiss, Germany).

### 2.7. Statistical Analysis

All data are presented as median ± standard error mean (SEM) and were analyzed using GraphPad Prism 6.0 software. Statistically significant differences between two groups were evaluated with the Mann–Whitney rank sum U test. *p*-values < 0.05 were considered significant.

## 3. Results

### 3.1. ADSCs from Patients with T2DM Have Enhanced Calcification after Osteogenic Differentiation

For the assessment of osteogenic potential, we induced ADSC osteogenesis with the consequent evaluation of calcification by Alizarin Red S staining. 

**Figure 1:**
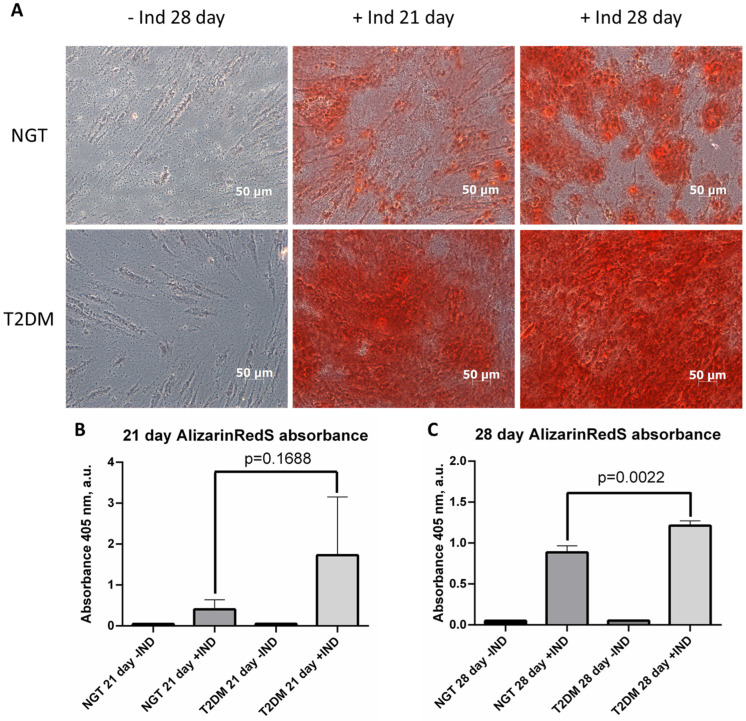
Morphology and calcification of ADSCs during osteogenic differentiation. (**A**)—representative images of Alizarin Red S staining of ADSC; (**B**)—spectrophotometric quantification of Alizarin Red S content in ADSCs on day 21 of osteogenesis; (**C**)—spectrophotometric quantification of Alizarin Red S content in ADSCs on day 28 of osteogenesis. The data are presented as median ± SEM; the Mann–Whitney rank sum U-test was used to calculate the significance of differences. Abbreviations: NGT—normal glucose tolerance; T2DM—type 2 diabetes mellitus; IND—osteogenesis induction; ADSC—adipose-derived stem cells; SEM—standard error mean.

Alizarin Red S staining showed that ADSCs from both NGT and T2DM individuals with obesity can differentiate in the osteogenic direction. However, ADSCs from the T2DM patients demonstrated significantly higher calcification levels than NGT patients on days 21 and 28 of differentiation (Figure 1A). This observation was confirmed by the spectrophotometric analysis of Alizarin Red S extracts from differentiated ADSCs. On day 21, a trend of increasing ADSC calcification from T2DM patients was observed (*p* = 0.1688; Figure 1B). On day 28, a statistically significant increase in ADSC calcification for T2DM patients (*p* = 0.0022; Figure 1C) was found. Thus, phenotypically T2DM ADSCs demonstrated significantly higher osteogenic potential than ADSCs from NGT patients.

### 3.2. RUNX2 mRNA Level Was Significantly Higher after Osteogenic Differentiation in T2DM ADSCs

The further estimation of ADSC osteogenesis required the analysis of osteogenic marker expression—the RUNX2 and SOX2 transcription factors and the SMAD4 signaling protein.

**Figure 2:**
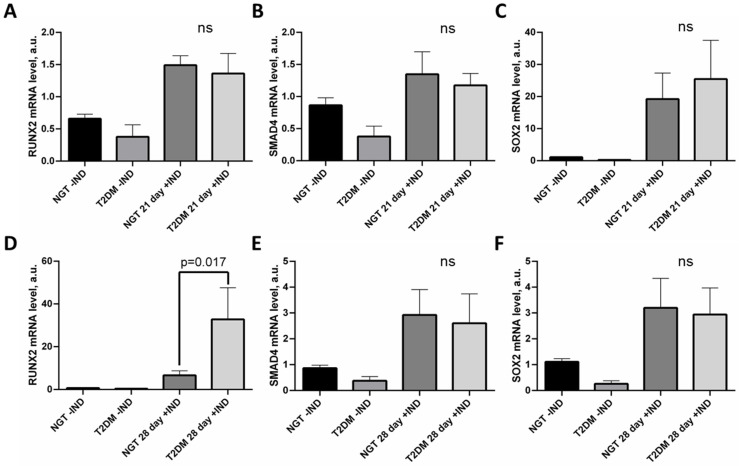
Expression of RUNX2, SMAD4, and SOX2 in ADSCs from NGT and T2DM patients during osteogenic differentiation. (**A**)—RUNX2 mRNA level on day 21; (**B**)—SMAD4 mRNA level on day 21; (**C**)—SOX2 mRNA level on day 21; (**D**)—RUNX2 mRNA level on day 28; (**E**)—SMAD4 mRNA level on day 28; (**F**)—SOX2 mRNA level on day 28. Data are presented as median ± SEM; the Mann–Whitney rank sum U-test was used to calculate the significance of differences. Abbreviations: ns—non-significant; NGT—normal glucose tolerance; T2DM—type 2 diabetes mellitus; IND—osteogenesis induction; ADSC—adipose-derived stem cells; SEM—standard error mean.

The expression of RUNX2, SMAD4, and SOX2 was activated in ADSCs under osteogenic conditions. On day 21, no statistically significant difference in osteogenic marker expression between NGT and T2DM groups was found (Figure 2A–C). On day 28, the expression of SMAD4 and SOX2 was equal (Figure 2E,F) in contrast to RUNX2, which was upregulated in ADSCs from T2DM patients (*p* = 0.017; Figure 2D). We suggest that the activation of osteogenesis and the calcification of ADSCs derived from T2DM individuals with obesity is associated with the enhanced expression of RUNX2.

### 3.3. ADSC from T2DM Individuals with Obesity Have Reduced Collagen I and Syndecan 1 Protein Expression 

To suggest a possible mechanism of osteogenesis activation in ADSCs from T2DM patients, we analyzed the expression of collagen I, which is important for normal osteogenesis, and syndecan 1, which supports adipogenesis [32,33,34].

**Figure 3:**
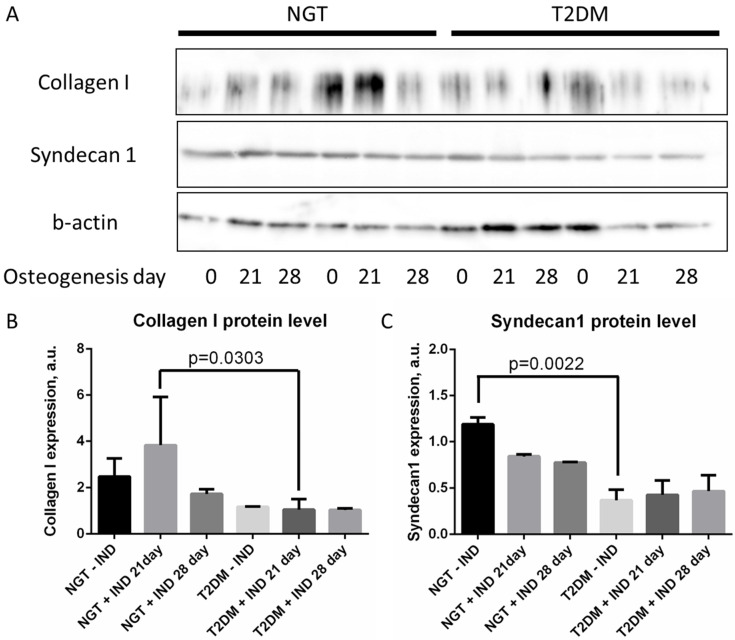
Protein expression of collagen I and syndecan 1 in ADSCs during osteogenesis. (**A**)—representative images; (**B**)—statistical analysis of immunoblot data for collagen I; (**C**)—statistical analysis of immunoblot data for syndecan 1. Data are presented as median ± SEM; the Mann–Whitney rank sum U-test was used to calculate the significance of differences. Abbreviations: NGT—normal glucose tolerance; T2DM—type 2 diabetes mellitus; IND—osteogenesis induction; ADSC—adipose-derived stem cells; SEM—standard error mean.

Investigating ADSCs at different stages of differentiation revealed an upward trend in the expression of both collagen I and syndecan 1 during osteogenesis in the NGT group but not in the T2DM group. In particular, collagen I protein expression was significantly higher on day 21 of ADSC osteogenesis for NGT patients (*p* = 0.0303; Figure 3A,B), and syndecan 1 protein expression was significantly higher in the ADSCs of NGT patients (*p* = 0.0022; Figure 3A,C) before osteogenesis in comparison to ADSCs from T2DM individuals. These data testify to the predisposition of ADSCs from NGT patients to adipogenesis and ADSCs from T2DM patients to osteogenesis.

### 3.4. Enhanced Osteogenic Potential Associates with Cell Senescence in ADSC of T2DM Patients

The senescence of stem cells is a process that disturbs their proliferation and differentiation potentials that has been observed in pathological states [35,36]. Accordingly, the cell senescence of ADSCs can be associated with the shifted differentiation potential of ADSCs from T2DM individuals.

**Figure 4:**
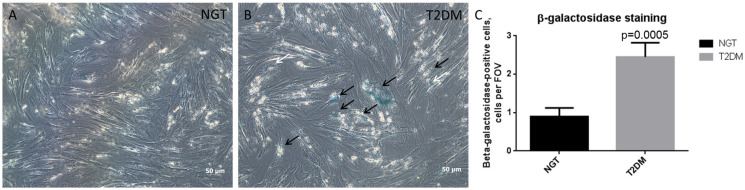
Enhanced osteogenesis of T2DM ADSCs is accompanied by cell senescence. (**A**)—representative image of NGT ADSCs after cell senescence assay; (**B**)—representative image of T2DM ADSCs after cell senescence assay; (**C**)—statistical analysis of staining. The data are presented as median ± SEM; the Mann–Whitney rank sum U-test was used to calculate the significance of differences. Abbreviations: NGT—normal glucose tolerance; T2DM—type 2 diabetes mellitus; ADSC—adipose-derived stem cells; SEM—standard error mean; FOV—field of view.

The analysis of cellular senescence revealed the significantly increased activity of beta-galactosidase in T2DM ADSCs, which reflects the enhanced cellular senescence that occurs during T2DM (Figure 4A–C). These data suggest the role of cellular senescence and associated cell cycle, metabolism, and secretome alterations in ADSC differentiation.

## 4. Discussion

In this study, we demonstrated the enhanced osteogenic potential of ADSCs from T2DM patients in comparison to NGT patients with respective changes of osteogenic markers mRNA expression, changes of ADSC-differentiation regulatory-factor expression, and enhanced cell senescence. Firstly, we discuss the physiological results of our study in the context of recent research. Recent studies of ADSC osteogenesis in T2DM conditions have been contradictory [23,24,25,26]. Studies on animal models of T2DM and hyperglycemia have shown impaired ADSC osteogenic potential [25,26]. However, a study on high-fat diet model of T2DM demonstrated the enhanced osteogenesis ability of T2DM ADSCs [23]. The study of Skubis-Sikora and co-authors [24] was highly similar to the present work: the investigation was performed on clinical material and included patients with obesity and T2DM. The analysis of this study showed that our results are mutually confirmed. It seems that for patient-derived ADSCs, the conclusion regarding enhanced osteogenesis in diabetic conditions remains appropriate. Moreover, a high-throughput study of miRNA composition of adipose tissue from patients with obesity demonstrated high pro-osteogenic and pro-fibrotic miRNA expression in the adipose tissue of T2DM patient [37]. However, adipogenesis and osteogenesis have reciprocal interactions: osteogenesis inhibits adipogenesis and vice versa [38]. Previously, we demonstrated a decreased ADSC adipogenic potential for T2DM patients in comparison to NGT patients [11]; this finding was confirmed by other authors [39,40]. In summary, these studies confirm that under diabetic conditions, the ADSCs of patients with obesity are committed to differentiation in the osteogenic lineage and have decreased adipogenic potential.

In gene expression analysis, we used three marker genes: RUNX2 as a crucial osteogenic transcription factor, SMAD4 as an intracellular transmitter of signal in the BMP4/TGFβ-dependent signaling pathway, and SOX2 as a stemness factor. RUNX2 expression was significantly higher in T2DM ADSCs at day 28 of osteogenesis. According to Alizarin Red S staining, this was expected because RUNX2 is the master regulator of ADSC osteogenic differentiation and calcification growth should be accompanied by RUNX2 activation [41,42]. Osteogenesis induction is driven by Wnt-dependent, NOTCH- and BMP4/TGFβ-signaling cascades, and the BMP4/TGFβ-signaling axis is crucial for osteogenesis [43,44,45]; however, SMAD4 expression during osteogenesis was found to be equal in the NGT and T2DM groups. Nevertheless, SMAD4 is a signaling molecule that can be regulated by phosphorylation, and the expression of SMAD4 gene may not be involved in the activation of osteogenic differentiation. Moreover, the induction cocktail did not contain specific activators of BMP4/TGFβ-signaling pathways.

SOX2 is a stemness factor that maintains a non-differentiated phenotype [46]. However, SOX2 also is known as a suppressor of osteogenic differentiation and an obligatory factor for adipogenesis [47,48]. In our study, SOX2 expression was similar at days 21 and 28 of osteogenesis in the NGT and T2DM groups. We suggest that the absence of effects on SOX2 expression indicates the absence of SOX2 participation in the regulation of adipo-osteogenic balance during obesity. Thus, among three important osteogenic factors, only RUNX2 is associated with the activation of ADSC osteogenesis during T2DM development in obese individuals.

Interactions between cells and the extracellular matrix strongly regulate differentiation process and commitment. We analyzed the expression of collagen I as an important factor of ADSC adipogenesis and osteogenesis [49,50]. In our study, we showed a general trend of enhanced collagen I expression during osteogenesis in NGT-derived ADSCs in comparison to the T2DM group. This observation is consistent with the decreased adipogenic potential of T2DM ADSCs reported previously [11,18]. The role of syndecan 1 in ADSC differentiation is also contradictory: on one hand, syndecan 1 is known to be a significant activator of adipogenesis [51,52], but, on the other hand, syndecan 1 can move ADSC differentiation potential towards osteogenesis [53]. Our protein expression analysis revealed the increased expression of syndecan 1 in ADSCs before osteogenesis in the NGT group. Our knowledge about reciprocal relationships between osteo- and adipogenesis suggests that the differentiation potential of T2DM ADSCs shifts toward the osteogenic direction.

Recent studies have shown the participation of cell senescence in the regulation of ADSC differentiation potential. The results of these studies remain unclear: some studies have reported the suppression of all differentiations [54,55], but some studies have demonstrated the enhanced osteogenic potential of senescent ADSCs [56,57]. The senescence-dependent enhancement of osteogenesis seems pathological: the senescence-associated secretory phenotype contains many pro-fibrotic, pro-inflammatory, and immunosuppressive soluble factors including TGFβ, which is a strong inducer of osteogenesis [58,59,60]. Our study revealed an increased level of cellular senescence in the ADSCs of T2DM patients in comparison to those of NGT patients, which may demonstrate the commitment of T2DM ADSCs to osteogenesis. In summary, we suggest that T2DM-associated cellular senescence can cause ADSC differentiation to shift toward osteogenesis, the impaired formation of new fat depots in adipose tissue, and the development of insulin resistance. The balance between ADSC adipo- and osteogenesis commitment is crucial for the determination of the metabolic fate of patients and adipose tissue. 

## 5. Conclusions

The purpose of this study was to analyze the osteogenic commitment of ADSCs from patients with obesity and with or without metabolic complications. We have shown that osteogenic shifts have critical impacts on the systemic metabolism of obese patients and predict the development of metabolic complications.

## 6. Limitations

The limitation of this study was its relatively small number of patients. Moreover, all participants of this study were women, though statistically significant differences were found in this patient sample set. 

## Figures and Tables

**Table 1:** Sequences of primers for real-time PCR.

Gene	Forward Primer	Reverse Primer
RUNX2	5′-TAGGCGCATTTCAGGTGCTT-3′	5′-GGTGTGGTAGTGAGTGGTGG-3′
SMAD4	5′-TGCATTCCAGCCTCCCATTT-3′	5′-TGTGCAACCTTGCTCTCTCA-3′
SOX2	5′-GCTTAGCCTCGTCGATGAAC-3′	5′-AACCCCAAGATGCACAACTC-3′
β-actin	5′-CGGTTCCGATGCCCTGAGGCTCTT-3′	5′-CGTCACACTTCATGATGGAATTGA-3′

## Supplementary Materials

The following supporting information can be downloaded at: https://www.mdpi.com/article/10.3390/life12050688/s1, Figure S1: Uncropped images for WB; Table S1: Characterization of patients which participated in the study.

## Abbreviations

ADSC—adipose-derived stem cells; BMP4—bone morphogenetic protein type 4; DMEM—Dulbecco’s Modified Eagle’s Medium; DNA—deoxyribonucleic acid; FBS—fetal bovine serum; HRP—horseradish peroxidase; mRNA—matrix RNA; NGT—normal glucose tolerance; NANOG—homeobox protein NANOG; OCT4—octamer-binding transcription factor type 4; OXPHOS—oxidative phosphorylation; PBS—phosphate buffer saline; PPARg—peroxisome-activated proliferator receptor type gamma; RUNX2—Runt-related transcription factor type 2; RT PCR—real-time polymerase chain reaction; SOX2—SRY-box transcription factor type 2; SDS PAGE—polyacrylamide gel electrophoresis with sodium dodecylsulphate; SEM—standard error mean; SMAD4—SMAD family member type 4; TGFβ—transforming growth factor type β; TBST—Tris buffer solution supplemented with Tween20; T2DM—type 2 diabetes mellitus.
