# Supplementary material for: Type 2 Diabetes Mellitus Facilitates Shift of Adipose-Derived Stem Cells Ex Vivo Differentiation toward Osteogenesis among Patients with Obesity
Source: Life (Basel). 2022 May 6;12(5):688. doi: 10.3390/life12050688 (PMC9146836; doi:10.3390/life12050688)
Supplement: Supplementary file 1 [file life-12-00688-s001.zip › life-1711380-supplementary.pdf]

life

MDPI

Article

Type 2 diabetes mellitus facilitates shift of adipose-derived stem cells differentiation toward osteogenesis among obese individuals

Margarita Agareva [1,2], Iurii Stafeev [2,*], Svetlana Michurina [2,3], Igor Sklyanik [4], Ekaterina Shestakova [4], Elizaveta Ratner [2], Xiang Hu [5], Mikhail Menshikov [2], Marina Shestakova [4] and Yelena Parfyonova [2,6]

Supplementary Table S1. Characterization of patients which participated in the studyю Data are presented as median ± SEM, Mann-Whitney rank sum U-test, statistically significance threshold p<0.05. N/A - non applicable.

Parameter	NGT	T2DM	p
Sex, female:male	3:0	3:0	N/A
Age, years	46±2.28	52±4.46	0.4307
Body weight, kg	118±9.6	113±3.2	0.5
BMI, kg/m2	45.84±0.85	40.18±1.42	0.0103
HOMA-IR, a.u.	5.99±1.97	5.89±1.03	0.9
M-index, mg/kg/min	5.35±0.62	1.44±0.12	0.0022
HbA1c, %	5.7±0.11	6.7±0.21	0.0022
FBG, mM	4.96±0.19	7.55±0.63	0.0017

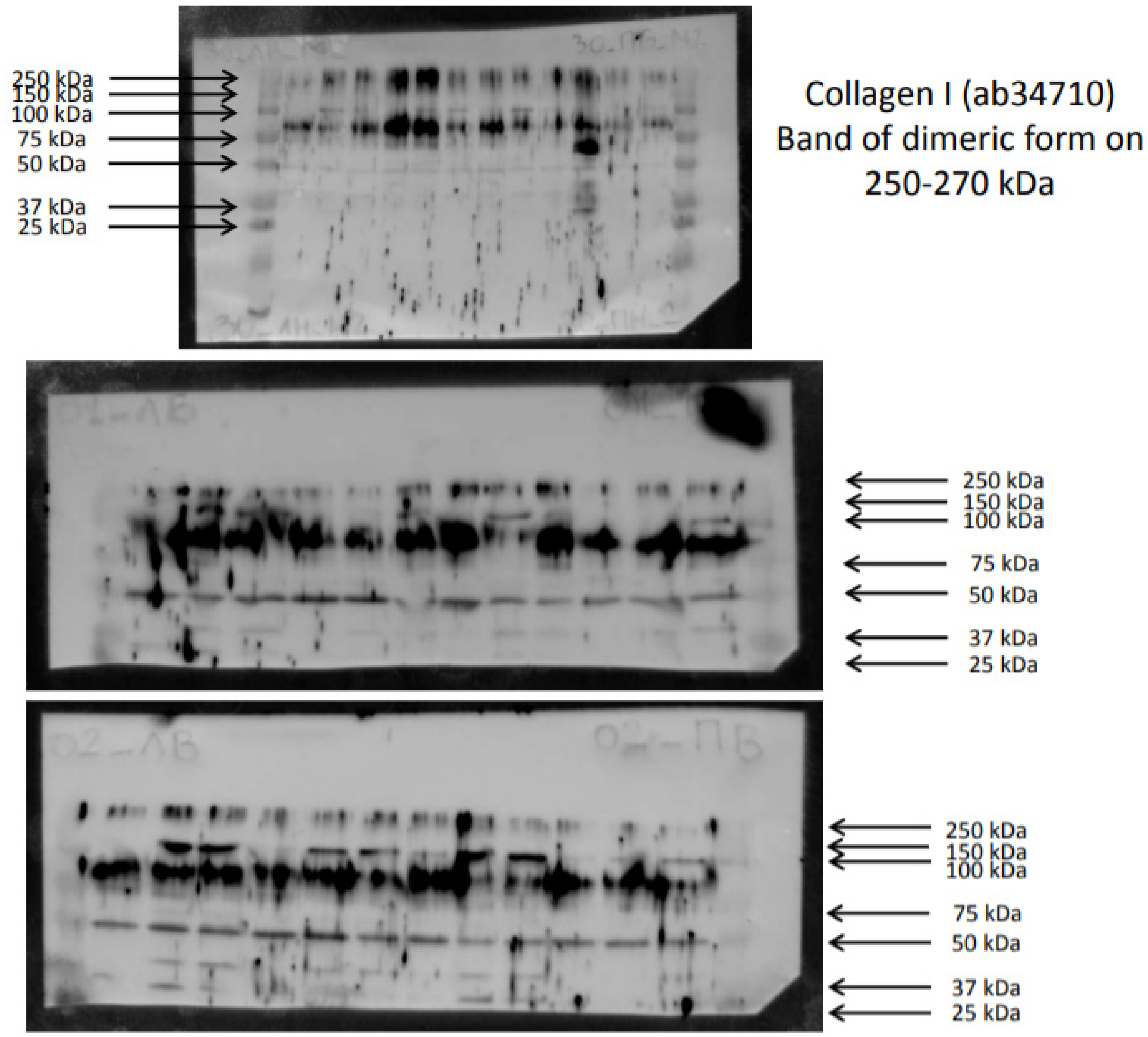

Figure S1. Uncropped images for WB.
